# No parts left behind? Exploring pros and cons of cooking whole chicken dishes at homes in Germany

**DOI:** 10.1016/j.psj.2025.106227

**Published:** 2025-12-09

**Authors:** Claire Siebenmorgen, Annik Spreckelmeyer, Micha Strack, Daniel Mörlein

**Affiliations:** a Department of Animal Sciences, University of Goettingen, D-37077 Goettingen, Germany; b isi GmbH, Institute for Sensory Research and Innovation Consulting, D-37124 Rosdorf, Germany

**Keywords:** Whole carcass, Consumer acceptance, Willingness to pay, Sensory evaluation, Sustainable meat consumption

## Abstract

Despite the growing popularity of alternative diets, meat consumption in Germany remains high. Poultry is particularly popular, with breasts and legs being the most in demand. Whole chickens, however, play a negligible role in marketing, which is problematic from an ecological perspective. This study examined the potential of whole-carcass marketing of female laying local chicken breeds in German households by means of a Home Use test. Overall, 108 participants prepared and evaluated two dishes, advised by a cooking kit, each with a different breed and all needed ingredients. While the home-cooked chicken soup received significantly higher overall liking scores than the chicken fricassee (*p* = .017), the chicken breed itself had no significant effect on overall liking. A consumer segmentation approach based on attitudinal items revealed that 56 % of participants showed a positive attitude toward using whole chickens, while 25 % found the preparation too time-consuming, and another 19 % expressed feelings of disgust. Household income, family structure, cooking skills additionally influenced both acceptance and willingness to pay. After an informational text, willingness to pay for local breeds did not increase. The findings provide valuable insights into consumer segmentation and highlight both opportunities and barriers to marketing of whole chicken in Germany.

## Introduction

In 2025, the chicken meat consumption in Germany and in the European Union (EU) remains at an all-time high and is still rising (BLE, 2024). As quantity increased, suppliers changed the form by which they supplied chicken parts over time. In the early 1990s, frozen, whole chickens made up to 80 % of the poultry supply in Germany (Buntzel and López, 2007). Today, this ratio is nearly inverted, with more than 80 % of the chicken meat now offered dissected into cuts (mostly breast and thighs) (DLG, 2024).

The preference for specific poultry cuts draws not only critical concerns regarding climate impact but also consequently affects economies and ecologies across the globe (European Commission, 2021). In particular, EU poultry production exports the “worthless” leftover parts greatly to countries in West Africa, skewing the markets. Cheap EU imports replace native poultry items there, which weakens smallholder incomes and endangers regional food independence (Anihouvi et al., 2024; Banson et al., 2015; Madibana et al., 2024; Rudloff and Schmieg, 2016; Zamani et al., 2021). The spread of both microbiomes and also zoonotic diseases, for example avian influenza, is eased through this international poultry “trade” (Anihouvi et al., 2024; Wu and Perrings, 2018). Greenhouse gas emissions are also increased as a result of transport for large volumes of poultry meat across long distances and enlarges the global environmental burden even more.

The German poultry industry is seeing simultaneously structural challenges to meet the high demand for standardized cuts (breast and thighs), with the industrial poultry farming relying on specialized high-performance breeds and hybrids (Augère-Granier, 2019; Magdelaine et al., 2008). In contrast to the modern hybrids, local chicken breeds, which have no high yielding fattening performance and often show a comparatively small proportion of breast meat, have been displaced nearly completely (Nolte et al., 2020). However, as a direct consequence to this market disappearance, the genetic diversity within the chicken population is rapidly decreasing (the so-called agrobiodiversity loss) (Mathew and Mathew, 2023; Sponenberg et al., 2019; Sukumaran and Keerthi, 2023). However, the preservation of agro-biodiversity is really important for further agricultural adaptation processes, since local chicken breeds frequently exhibit particular attributes like high resilience to local climates, disease resistances, and are more adaptable to free-range organic systems (Augère-Granier, 2019; Bettridge et al., 2018; Lyimo et al., 2014). Therefore, the conservation of these local chicken breeds provides benefits by maintaining a larger pool of genetic resources, which serves as a safeguard and is essential for ensuring long-term food security (Poore and Nemecek, 2018).

In Germany, several local chicken breeds hold considerable historical and cultural significance. The Bielefelder Kennhuhn (BIE), developed in 1976 in Hanover, is known for its high growth performance and is currently listed as not endangered. In contrast, the Altsteirer (ALT), which originated in the 13th century in the region of southern Germany and northern Austria, and the Ramelsloher (RAM), first bred in 1874 in northern Germany, are classified as critically and extremely endangered, respectively, according to the German Federal Agency for Agriculture and Food (BLE, 2023).

A promising approach for conserving (endangered) local chicken breeds is the targeted direct marketing of whole animals. By creating value for the entire carcass, not only for the highly demanded breast and thigh meat but also less-preferred parts such as wings, necks, and carcasses, the overall economic viability of these breeds increases, while at the same time the export of low-priced leftovers that can undermine smallholder markets in Africa can be reduced. In a broader perspective, conserving agrobiodiversity actively also requires adapting consumption habits: eating less chicken meat in total, but choosing products from responsible local sources, preferred in line with the principle of “less but better.”

Yet, there is no scientific evidence how to manage such a goal. What are the factors that promote or inhibit the use of whole chickens today by private households in the EU? This study therefore aimed to assess the opportunities and barriers for using whole chickens of local chicken breeds in the context of German private households. It explored how differences in breed and preparation method affect palatability and overall appeal, and how these, in turn, influence the value people place on locally produced whole chickens. Beyond the German context, the methodological approach and insights of this study may contribute to efforts in promoting sustainable livestock use and agrobiodiversity conservation in other parts of the world.

## Material and methods

In the present study, a Home Use test (HUT) was conducted with untrained consumers. Over the course of two consecutive weeks, participants each received two chickens originating from three local breeds; commercial chickens were used as a control. Participants were not informed about the breed type or origins of the chickens. With every chicken a recipe and ingredients were provided such as participants could prepare dishes (chicken soup and chicken fricassee). Via an online questionnaire, questions regarding sensory appeal, attitudes and demographics were assessed.

### Ethics

The experiment was conducted in compliance with applicable national and international regulations, including EU Directive 2010/63. All procedures involving animals adhered to principles of Good Veterinary Practice. Ethical approval of the animal experiment was granted by the competent German authority of Lower Saxony (file number 33.19-42502-04-00-00204). Ethical approval for the consumer study was granted by the Ethics Committee of the University of Goettingen on January 30, 2024.

### Selected animals

#### Local breeds

For the experiment laying-hens from three traditional German female chicken breeds BIE, ALT, and RAM were selected. These breeds were chosen due to their inclusion in the project “ÖkoGen” (BLE, 2022). The project aims to promote the preservation of local German chicken breeds by increasing their populations and evaluating their potential as dual-purpose chickens that contribute to the conservation of agrobiodiversity. The chickens hatched at the University of Bonn and were reared under organic conditions in accordance with the EU Regulation (EU) 2018/848. Stocking density was at 8 animals per m² in indoor area, and 4 m² of outdoor run per bird. Birds had access to the outdoor area for a minimum of 8 h per day, weather permitting. The house was naturally lit and supplemented with artificial light, as well as temperature regulated with 18–20°C on average. The floor was covered with organic straw litter and equipped with pecking and scratching materials to stimulate natural behavior. Feed and water were provided ad libitum. They were fed age-specific organic feedstuff (Curo Spezialfutter GmbH, Ennigerloh, Germany), including broiler starter pullet mashes (namely Maxima 3-9 and 9-17), and layer mash (namely Optima 1 OR). From week 16 onward, laying nests were provided. At the age of one year, the hens were transferred to the poultry research facilities of the Department of Animal Sciences at the University of Goettingen, where they were kept as laying hens for another six months. Their diet consisted mainly of Curo Bio Lege Allein (Curo Spezialfutter, Ennigerloh, Germany), supplemented with environmental enrichment such as alfalfa briquettes, pecking stones, mixed grains, grit, and straw. Community nest boxes with straw bedding with one nest per seven hens were used. Mortality during the laying period was below 5 %, mainly due to natural causes. Birds were vaccinated according to standard organic poultry health management protocols, including vaccinations against Marek’s disease, infectious bronchitis, and Newcastle disease. The hens exhibited average laying performances of 38.4 % (BIE), 27.1 % (ALT) and 34.1 % (RAM). All birds were slaughtered at the end of their laying period (16 months of age) at Bio Frischgefluegel Roth GmbH & Co. KG (Witzenhausen, Germany). Electrical stunning and blood drainage was conducted according to EU regulations. The average slaughter weights are shown in Table 1. After evisceration the whole chickens were chilled at +4 °C for 24 h. They were then vacuum-sealed and transported to the Laboratory for Sensory Analysis and Consumer Research at the University of Goettingen, where they were frozen and stored at –21 °C for six months until the experiment was conducted.

**Table 1:** Average carcass weight ± standard deviation (SD) after slaughtering.

Slaughterweight (g)	BIE	ALT	RAM	STD
Mean ± SD	1590 ± 285.2	1388.6 ± 269.2	1348.3 ± 229.5	1145.6 ± 82.2

BIE, Bielefelder Kennhuhn; ALT, Altsteirer; RAM, Ramelsloher; STD, Lohmann Brown.

#### Standard breeds

As a control group, standard hybrid laying hens of the breed Lohmann brown (STD) were commercially sourced from the farm Werderhof (Goettingen, Germany) with their average carcass weight shown in Table 1. The animals had been slaughtered at the age of 16 months at the same slaughterhouse following the same procedure as the local breeds. The carcasses were also frozen at −21°C until the experiment was conducted.

### Participants

For the experiment a total of *n* = 120 were intended, but only *n* = 117 participants were recruited. Data cleaning was performed to ensure validity and consistency of the responses. Questionnaires that were not fully completed as well as cases in which the dishes were not mainly prepared according to the provided recipes (lacking photographic evidence) were excluded. The final cleaned dataset included *n* = 108 participants. Recruitment was carried out via an online screening questionnaire programmed on the platform EyeQuestion® (Version 5.2, Elst, Netherlands), which was accessible through a QR code. The code was distributed on flyers and posters at various locations of the University of Goettingen, as well as in supermarkets, kindergartens, and specialty food stores throughout the city. The screener ensured the selection of eligible participants based on the regular consumption of poultry meat. Upon successful registration, participants received an email invitation to pick-up a standardized cooking kit at the Laboratory for Sensory Analysis and Consumer Research (Goettingen, Germany) in two consecutive weeks. All participants gave their consent prior to participation and agreed to the experiment’s terms, including data collection and protection regulations in accordance with the EU standards. At the first pickup appointment, participants signed a consent and data privacy agreement. They also provided their bank details to enable the transfer of the participation incentive. Participation in the study was voluntary. Participants who collected both cooking bags and completed both questionnaires in full received a compensation of €15 per person. The incentive was provided to encourage full participation and ensure high response quality throughout the two-week study period.

### Prepared dishes

**Fig. 1:**
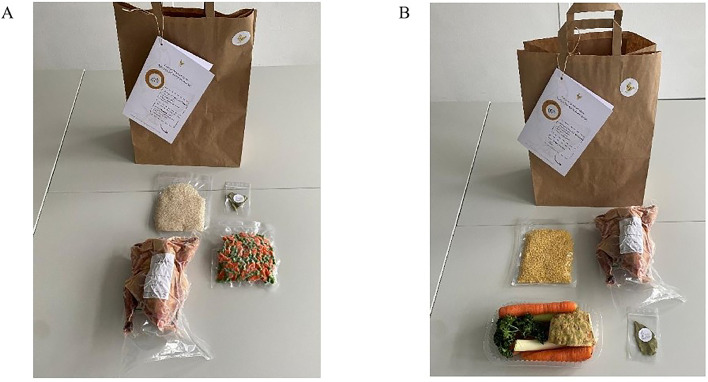
Cooking bags for the Home Use test with recipe and ingredients for (A) chicken fricassee and (B) chicken soup.

**Fig. A.1:**
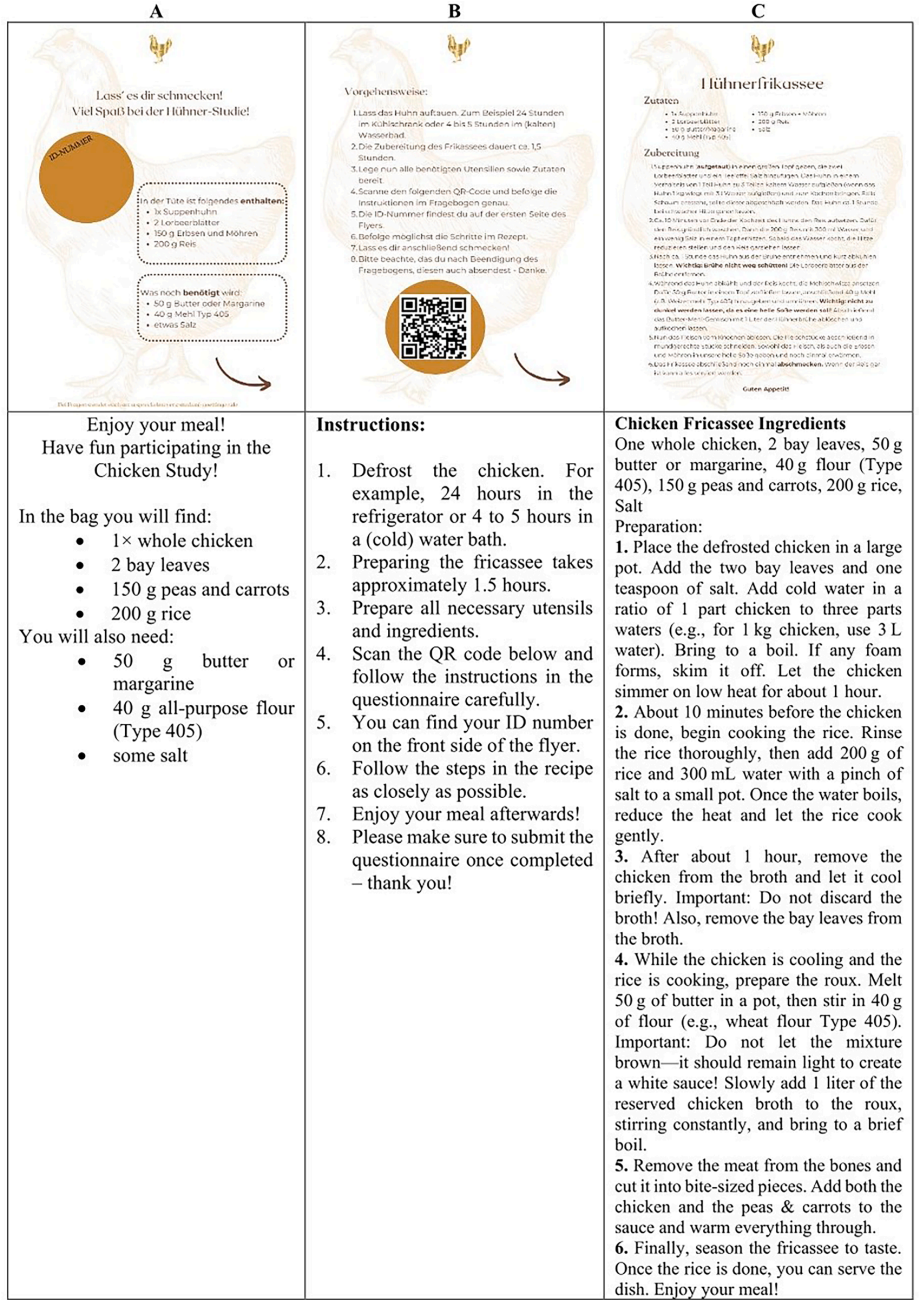
Recipe book for chicken fricassee including (A) title page with information on the contents and ingredients still needed, (B) general procedure of the experiment, as well as QR code for the questionnaire, and (C) recipe with ingredients and step-by-step explanation.

Participants prepared two predefined traditional German dishes: chicken soup and chicken fricassee. Participants were encouraged to follow the provided recipes as closely as possible to ensure consistency in preparation and evaluation (Fig. A.1, A.2). Because this was an in-home field experiment aimed at capturing real-world consumer behavior, recipe modifications were expected and purposefully documented as part of natural preparation practices. The ingredients were distributed in two standardized cooking bags, each containing all the necessary foodstuffs. Each cooking bag included a frozen whole chicken and the specific ingredients necessary for the assigned dish (Fig. 1). For the chicken soup, the kits contained two whole bay leaves (Kotanyi Orient GmbH, Austria), one pack of fresh soup vegetables (“Mirepoix”), including leek, celeriac, carrot, and parsley (Pfalzmarkt eG, Germany), as well as 175 g of vacuum-packed star-shaped soup noodles (Mamma Lucia, Eterna Nahrungsmittel GmbH, Germany). For the chicken fricassee, participants received two whole bay leaves, 150 g of vacuum-packed frozen peas and carrots (D'Arta, Belgium), and 200 g of vacuum-packed basmati rice (Mueller’s Muehle GmbH, Germany). A total of 240 cooking bags were prepared, 120 per distribution date, with 60 portions of each dish provided per week.

### Experimental design

The study adopted a HUT to assess the potential for the utilization and marketing of whole chicken breeds in private households. HUTs are affective-sensory consumer tests conducted in naturalistic settings, allowing untrained participants to evaluate food products in their own kitchens (Lawless and Heymann, 2010). An Incomplete-Block Design (factor: breed type) was combined with a Within-Subject Design (factor: type of dish). Not all participants received all four available chicken breeds. Instead, each participant received only a randomized subset of two breeds, ensuring balanced distribution across blocks. For each breed, 60 chickens were randomly assigned to the different blocks. The Within-Subject component ensured that each participant prepared and evaluated the two dishes and two different breeds. This design enabled the direct comparison of individual sensory impressions and preferences across different breeds and preparation methods (Charness et al., 2012). The allocation of dishes was randomized: half of the participants received the ingredients for chicken fricassee first, and the other half received those for chicken soup first. In the second week, the dishes were switched, ensuring that all participants prepared both recipes by the end of the study. This resulted in a total of 216 evaluations (108 soups and 108 fricassees) distributed across four breeds from *n* = 108 participants (Table 2). Deviations from perfect balance were due to participant dropout rather than allocation bias.

**Table 2:** Distribution of evaluated chickens across breeds and dishes from *n* = 108 participants.

Breed	Soup (n)	Fricassee (n)	Total (n)	% of total
ALT	47	16	63	29.2 %
BIE	8	38	46	21.3 %
RAM	13	39	52	24.1 %
STD	40	15	55	25.5 %
Total	108	108	216	100 %

BIE, Bielefelder Kennhuhn; ALT, Altsteirer; RAM, Ramelsloher; STD, Lohmann Brown;.

### Questionnaire

**Fig. A.3:**
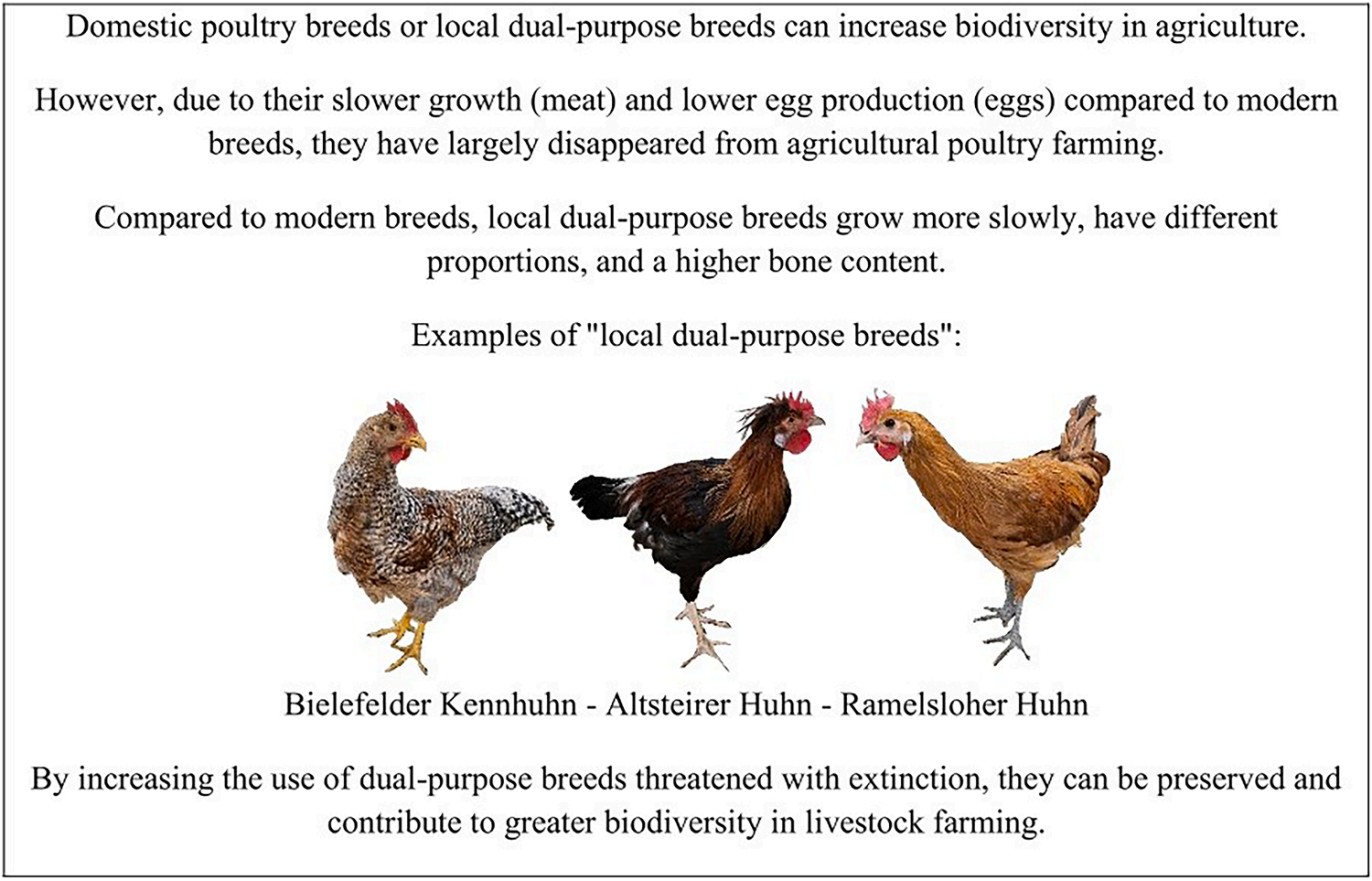
Integrated information treatment in the second questionnaire, with information on biodiversity, different body shapes and endangered breeds used in the HUT.

Two questionnaires were developed for the HUT which participants were required to complete each time while preparing one of the two assigned dishes. The questionnaires were created using EyeQuestion® and made accessible to participants via a QR code printed on the recipe sheets (Fig. A.1, A.2). Both questionnaires focused on the sensory characteristics of the chicken before, during, and after cooking, followed by an evaluation of the entire dish. Sensory attributes were assessed using a 9-point hedonic scale (1 = ‘dislike extremely’; 5 = ‘neither dislike nor like’; and 9 = ‘like extremely’), 5-point Just-About-Right (JAR) ratings (e.g., 1 = ‘too big’; 3 = ‘just right’; 5 = ‘too small’), as well as Check-All-That-Applies (CATA) questions. The questionnaire from week 1 additionally collected information on participants' chicken eating and cooking habits, as well as statements reflecting household poultry consumption behavior (13 attitudinal questions; see Table A.1). Further, some general food purchasing criteria and prior knowledge of local chicken breeds was asked. These questions were presented in multiple-choice format, along with questions capturing the willingness to pay (WTP) for local chickens and ranking tasks. The questionnaire distributed in week 2 mirrored the first in terms of sensory assessment. Furthermore, participants were asked about their connection with agriculture, knowledge of production practices, and familiarity with local chicken breeds. In the last part of the second questionnaire, participants received an informational treatment designed to raise awareness of the local breeds used in the study and the broader issue of agrobiodiversity loss (Fig. A.3). Following this intervention, participants were again asked about their WTP. Finally, sociodemographic data were obtained.

### Statistical analysis

Statistical analysis was conducted using IBM SPSS Statistics (Version 29, IBM Corp., Armonk, NY, USA). Initially, descriptive statistics including frequencies and percentages were used to characterize the sample. To reduce the dimensionality of attitudinal variables, a Principal Component Analysis (PCA) with Varimax rotation was conducted. Ipsatization was applied by mean-centering each participant’s responses across all attitudinal items in order to correct for individual response styles and to analyze relative agreement. Based on the dimensions of the PCA, participants were segmented hands-on into three distinct attitude groups. Segmentation was conducted by interpreting the orientation of individual factor scores within the two-dimensional PCA space (PC1 vs. PC2), visually identifying cluster-like regions corresponding to coherent attitude patterns, and assigning participants to the nearest segment boundary as defined by the PCA axes. This approach follows common practice in exploratory attitude research when clusters are already clearly represented in the PCA plot. Pearson correlations were used to explore associations between attitudes and personal characteristics. Correlation coefficients of *r* ≥ .16 were interpreted as marginally relevant (*p* < .10), and *r* ≥ .19 as statistically significant (*p* < .05). To test the influence of chicken breed and dish type on sensory ratings and practical suitability, linear mixed models were employed. Breed (ALT, BIE, RAM, STD) and dish (coded as +1 = chicken soup, –1 = chicken fricassee) were entered as fixed factors, and participants as a random effect. The interaction of the fixed factors was also included. Significance was determined at *p* < .05. Additionally, Chi² tests were used to examine the distribution of attitude segments across breeds and dishes. To identify predictors of willingness to pay (WTP) for local chicken breeds, forward-selection Ordinary Least Square regressions were performed. The models included income, price sensitivity, cooking behavior (e.g., recipe modifications), sensory experience, and demographic variables (e.g., gender, age). Based on these regressions, a path model was constructed to visualize the relationships between variables. Overall, the analytical approach combined descriptive, univariate, multivariate, and inferential methods. Mixed models accounted for individual variability in HUT responses, while regression analyses identified key drivers of consumer willingness to pay for local breeds.

## Results

### Participants, habits and attitudes

#### Sample characteristics

The 108 participants showed a slight overrepresentation of women (56.5 % female). The age distribution was found to be skewed towards younger adults, with 55.6 % aged between 25 and 39 years, and only two participants over the age of 65 (Table 3). Regarding household composition, 61.1 % lived in one- or two-person households, and 79.6 % reported living without children. The sample was highly educated, with nearly 51 % holding a university degree and 16.7 % holding a doctoral degree. About one third of the participants were employed full-time, and another third were students. The net household income exceeded €3,600 in 27 % of cases. From the 108 participants 27 % had no connection to agriculture, while 73 % reported at least one type of agricultural affiliation (e.g., buy locally from farms or farm shops, keeping livestock or work/study-related connections to agriculture)

**Table 3:** Sociodemographic characteristics of participants in the Home Use test (*n* = 108).

Question	Category	n	%	German federal statistics (%)
Gender	Female	61	56.5 %	50.7 %
	Male	45	41.7 %	49.3 %
	No response	2	1.9 %	-
Age	18-24 years	20	18.5 %	7.3 %
	25-39 years	60	55.6 %	19.2 %
	40-64 years	26	24.1 %	34.4 %
	65 years and older	2	1.9 %	22.3 %
Number of persons per household	1	23	21.3 %	41.1 %
	2	43	39.8 %	33.2 %
	3	15	13.9 %	12.0 %
	4	16	14.8 %	9.5 %
	5 or more	11	10.2 %	3.0 %
…of which children	0	86	79.6 %	Childless couples: 54.3 %
	1	12	11.1 %	42.5 %
	2	7	6.5 %	42.6 %
	3	2	1.9 %	3 or more children 14.9 %
	4	1	0.9 %	-
Education level	Compulsory school	1	0.9 %	28.6 %
	Vocational training	10	9.3 %	46.6 %
	High school diploma	24	22.2 %	33.5 %
	University degree	55	50.9 %	4.4 %
	Doctorate	18	16.7 %	1.2 %
Employment status	Full-time	36	33.3 %	55.4 %
	Part-time	27	25.0 %	13.9 %
	Unemployed	1	0.9 %	2.8 %
	Retired	2	1.9 %	21.9 %
	Student	38	35.2 %	3.3 %
	Other employment	4	3.7 %	2.7 %
Net household income	≤ €599	18	16.7 %	0.7 %
	€600-1,199	16	14.8 %	10.6 %
	€1,200-1,799	8	7.4 %	15.9 %
	€1,800-2,399	14	13.0 %	20.7 %
	€2,400-2,999	11	10.2 %	25.9 %
	€3,000-3,599	12	11.1 %	15.9 %
	> €3,600	29	26.9 %	10.3 %
Agricultural background	Non	29	27 %	33 %
	Friends/Relatives in agriculture	35	32 %	-
	Buy locally from farms/ farm shops	16	15 %	15 %
	Keep own livestock	7	6 %	-
	Work/Study-related connections	21	19 %	2 %

#### Cooking habits and purchasing behavior

In terms of cooking habits, the majority of participants (89 %) reported learning recipes primarily from online resources, followed by cookbooks (56 %) and videos on social media platforms such as Instagram or TikTok (39 %). Regarding hot meal preparation, 38 % cooked daily, 35 % at least three times per week, and 18 % two to three times per week. Food purchasing decisions were mainly driven by taste (82 %), perceived health quality (76 %) and regionality (46 %), while packaging avoidance (31 %), and organic origin (7 %) played smaller roles. During food purchasing, the type of chicken cut plays a significant role in participants preferences. Chicken breast was the most favored option (62 %), followed by wings (16 %) and thighs (14 %). Whole chickens were preferred by only 6 % of participants, while offal ranked lowest (2 %).

#### Condition sizes and correlations with whole-chicken use

**Fig. 2:**
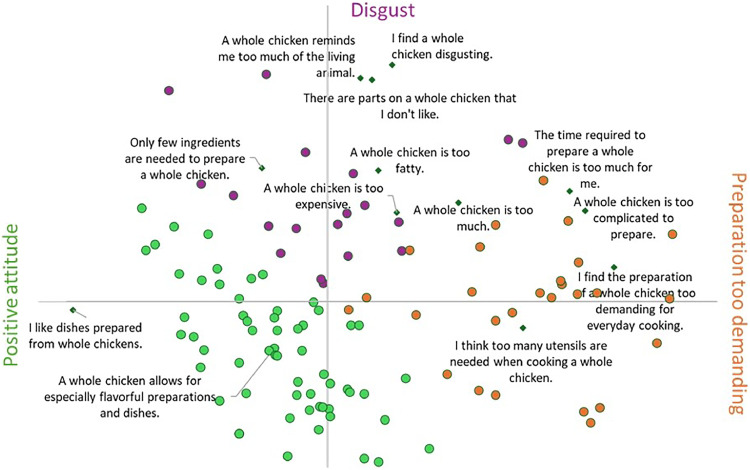
PCA of ipsative ratings on the perception of whole chickens, each point a participant, divided into three segments, *n* = 108.

To evaluate associations with whole chicken consumption, an inverted ranking score for the cuts was calculated. Correlation analyses with sociodemographic and behavioral variables revealed weak patterns (Table 4). The correlations were small in magnitude, indicating only limited associations, and suggesting that none of the examined variables showed a practically meaningful relationship with the preference for whole chickens. Participants living in households with children (*r* = .180) and those with higher net income (*r* = .178) were more likely to favor whole chickens. A trend was also observed for age (*r* = .128). Cooking knowledge and purchasing criteria appeared largely unrelated to preferences for whole chickens. However, participants who perceived cooking as more difficult (*r* = –.108) generally found the preparation of a whole chicken less feasible. Responses to 13 attitude statements (Table A.1; Fig. 2) allowed participants to be divided into three segments regarding whole chicken usage: "Positive attitude" (56 %), "Preparation too demanding" (25 %), and "Disgust" (19 %). The "Positive attitude" segment, for example, agreed with the statement "A whole chicken allows for the preparation of flavorful dishes". Phrases such as "I find the preparation of a whole chicken too demanding for everyday cooking" or "I think too many utensils are needed when cooking a whole chicken" are agreed by members of the " Preparation too demanding " segment. Statements such as " A whole chicken reminds me too much of the living animal" or "I find a whole chicken disgusting" fell into the "Disgust" segment.

**Table 4:** Correlations between sociodemographic and behavioral variables with the preference for whole chicken (*n* = 108).

Variable	Whole chicken preference (Inverse Rank Score)
Female	.055
Age	.128
Household size	.091
Children in household	.180*
University degree	-.107
Net household income	.178*
Agricultural background	.008
Price vs. regional preference	-.111
Health vs. convenience preference	-.077
Shelf life vs. husbandry	-.072
Origin vs. organic	.001
Hot meals per week	.037
Cooking knowledge: family/friends	-.111
Cooking knowledge: websites/blogs/books	.055
Cooking knowledge: cooking shows	-.031
Self-assessed cooking skills	.037
Perceived cooking difficulty	-.108
High frequency chicken consumers	-.073

Pearson correlation coefficients, r_crit_ .16 p_2t_ < .10*, r_crit_ .19 p_2t_ < .05**.

#### Sociodemographic correlates of the segments

**Fig. 3:**
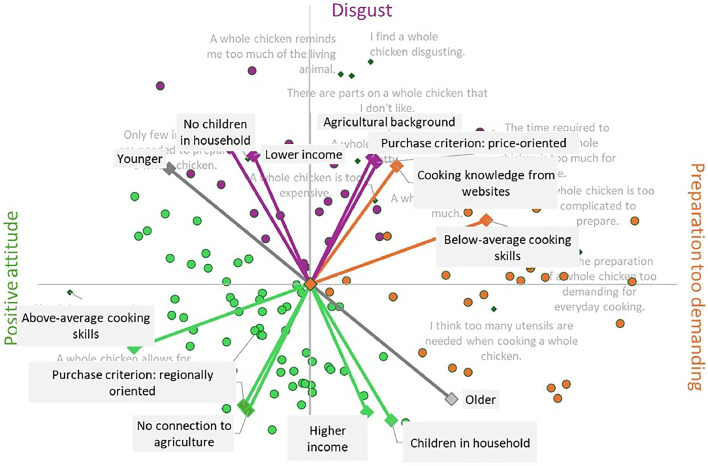
PCA of ipsative ratings with correlations of sociodemographic and behavioral characteristics, *n* = 108.

Furthermore, Fig. 3 shows correlations between sociodemographic and behavioral characteristics and the three attitude segments. Younger participants with low incomes, no children in the household, and high price sensitivity are more frequently represented in the "Disgust" segment. The " Preparation too demanding" segment is characterized by individuals with low self-assessment of their cooking skills and a high level of digital information use. In contrast, the "Positive attitude" segment is characterized by individuals with high cooking skills, above-average income, a preference for regional products but no connection to agriculture**.**

### Sensory evaluation

#### Effects of chicken breed

Randomization of breeds to participants and dishes resulted in varying condition sizes (expected *n* = 27), ranging from only 8 soups of BIE to 40 soups of STD and from 15 fricassees of STD to 39 fricassees of RAM (Table 5). Nevertheless, the mixed model is able to estimate the effects of the independent variables "chicken breed" and "dish" on the six dependent sensory variables. The BIE breed initially performed significantly better in terms of suitability for everyday usage than the RAM breed (*p* = .036). Attempts were made to explain this breed effect by chicken weight, but weight had no significant influence on suitability for everyday use. However, by including the three attitude segments ("Positive attitude" towards whole chickens, "Preparation too demanding", and "Disgust") the previously significant breed effect disappeared. This was because the segment with a generally positive attitude, who also rated everyday usage higher, received the BIE breed slightly more often and the RAM breed slightly less often than the other two segments. Thus, the observed breed effect appears to be a statistical artifact. All other five dependent variables showed no meaningful breed differences in consumer liking within the scope and power of this field study. The absence of further breed differences indicates that sensory evaluations were highly similar across breeds.

**Table 5:** Descriptives on a 9-point scale (raw Means (M) and standard deviation (SD)) for the Breed x Dish conditions (dish varied within participant).

Dish	Breed	Number	Odor raw chicken		Odor cooked chicken		Overall liking		Taste cooked chicken		Recook		Everyday usage	
		n	M	SD	M	SD	M	SD	M	SD	M	SD	M	SD
Fricassee	ALT	16	6.3	1.3	6.6	1.4	6.8	1.4	6.8	1.5	5.3	2.6	5.8	3.1
	BIE	38	6.3	1.4	7.1	1.3	6.9	1.8	6.7	1.8	6.1	2.7	5.9	2.5
	RAM	39	5.7	1.5	6.4	1.7	6.7	1.2	6.8	1.2	5.9	2.2	4.6	2.1
	STD	15	6.1	1.4	6.9	1.1	6.6	1.9	6.7	2.2	5.7	2.9	5.5	2.4
Soup	ALT	47	6.0	1.4	6.7	1.4	7.1	1.6	7.1	1.5	6.9	2.4	6.1	2.8
	BIE	8	6.3	1.2	6.8	1.0	7.8	0.9	7.9	0.8	7.9	1.0	7.4	1.2
	RAM	13	6.2	1.3	6.4	1.4	7.0	1.0	6.9	1.0	6.4	2.0	5.2	2.7
	STD	40	6.2	1.4	6.7	1.6	7.3	1.2	7.2	1.2	7.1	2.2	6.7	2.2

ALT, Altsteirer; BIE, Bielefelder Kennhuhn; RAM, Ramelsloher; STD, Lohmann Brown;.

#### Effects of dish type

In contrast, the type of dish significant influenced consumer likings. The chicken soup was rated higher than chicken fricassee in terms of overall liking (*p* = .017), taste liking (*p* = .009), willingness to cook again (*p* = .001), and suitability for everyday usage (*p* = .001) (Table 5, Table 6). To further explore the influence of the dish, two additional factors were considered in the analysis.

**Table 6:** Results of the initial mixed model with fixed factors breed and dish and random factor consumer ID on the six sensory dependent variables, *n* = 108.

Sensory variable / *p*-value	Breed	Dish	Breed x Dish	Means (9-point scale)
Odor raw chicken	n.s.	n.s.	n.s.	Fricassee 6.1, Soup 6.2
Odor cooked chicken	n.s.	n.s.	n.s.	Fricassee 6.7, Soup 6.7
Overall liking	n.s.	.017*	n.s.	Fricassee 6.7, Soup 7.3
Taste cooked chicken	n.s.	.009**	n.s.	Fricassee 6.8, Soup 7.1
Recook	n.s.	.001***	n.s.	Fricassee 5.9, Soup 6.9
Everyday usage	.036*	.001***	n.s.	Fricassee 5.3, Soup 6.3

Significant effects are indicated for *p* < .05 (*), *p* < .01 (**), and *p* < .001 (***). n.s., not significant (*p* > .05).

#### Influence of recipe and meat-removal modification

Firstly, 40 % of participants reported they had modified the recipe by adding additional ingredients (mostly spices). This group of participants rated the dishes better (overall liking, taste liking, recooking *p* < .05), especially the overall liking of the fricassee increased with recipe modification (*p* = .05). Secondly, the way participants removed meat from the bone had a notable impact on their evaluations. In total, 47.2 % removed the chicken meat from the carcass by hand, 37.5 % used tools such as a knife or fork, and 15.3 % used both methods. How the meat was removed significantly affected the overall liking (*p* < .001), taste (*p* < .001), and the willingness to recook (*p* < .001). For chicken fricassee, the average overall liking was *M* = 7.4 (*SD* = 1.2) when the meat was removed by hand, and *M* = 6.0 (*SD* = 1.5) when utensils were used. A similar pattern was observed for the overall liking of the chicken soup, with hand removal resulting in *M* = 7.4 (*SD* = 1.1) and utensil use in *M* = 6.8 (*SD* = 1.3). These differences illustrate a clear and consistent advantage of hand removal in terms of consumer liking.

#### Perceived size and carcass weight

**Fig. 4:**
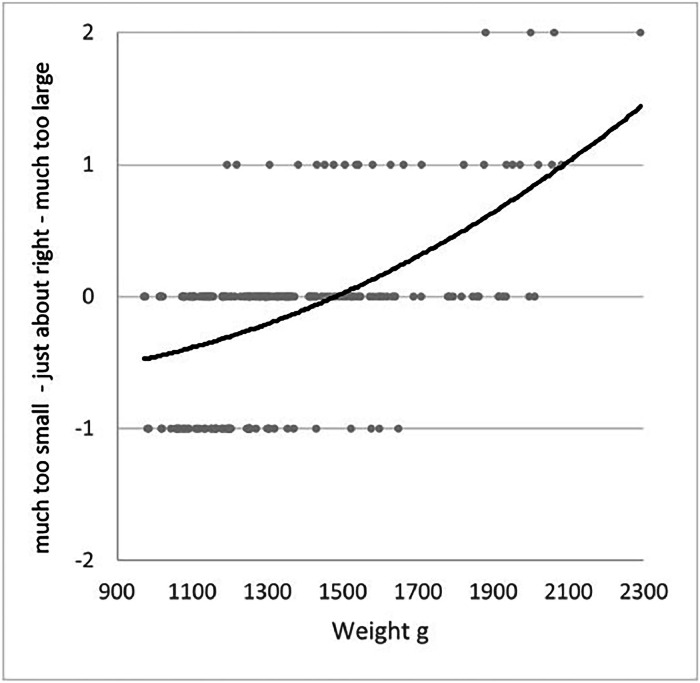
Quadratic relationship between carcass weight (g) and perceived size of the chicken, based on Just-About-Right (JAR) ratings, *n* = 2 × 108.

Furthermore, participants rated the perceived size of the chicken using a JAR scale. The responses revealed a quadratic relationship between carcass weight and perceived size (Fig. 4). Chickens with medium weights (approximately 1300 - 1700 g) were frequently rated as “just about right”, whereas lighter chickens (for example the STD) were often perceived as “too small”, while heavier carcasses (e.g., upper-range BIE) were more frequently rated as “too large”. Carcass weight had a statistically significant effect on the perceived raw-odor intensity (*p* = .041), with higher ratings at greater weight. This effect was modest but indicates a slight increase in odor intensity with increasing carcass size. No other sensory attributes were significantly affected by differences in carcass weight (*p* > .05).

### Willingness to pay

**Fig. 5:**
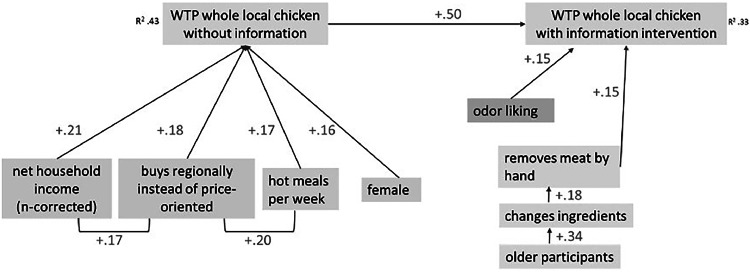
Path model with standardized regression coefficients on willingness to pay (WTP) from the first dish (no information) and second dish (with information), *n* = 108.

The WTP for a whole chicken of a local breed was analyzed and presented as a path model using standardized regression analyses (Fig. 5). From the questionnaire provided with the first dish, WTP was assessed without any additional information about the local chicken breeds (*R²* = 0.43; Fig. 5, left). After an informational treatment on agrobiodiversity loss, WTP was again assessed in the second questionnaire handed out in week 2 (*R²* = 0.33; Fig. 5, right). The model quantifies the influence of various factors using standardized regression coefficients (*β*-values), where higher values indicate stronger associations. WTP increased with rising income (*β* = +0.21). Likewise, lower price sensitivity (*β* = +0.18), higher frequency of cooking warm meals per week (*β* = +0.17), and being female (*β* = +0.16) were all associated with a higher willingness to pay. Naturally, the price stated after the second dish and the informational treatment was strongly associated with the initial price given after the first dish (*β* = +0.50). In addition, WTP increased with the pleasantness of the perceived smell of the chicken during the home-use test (*β* = +0.15), and when the meat was removed from the bone by hand (*β* = +0.15). The WTP, is furthermore positively related to the modification of the original recipe by additional ingredients (*β* = +.18). Overall, these values indicate several modest but consistent predictors of willingness to pay.

## Discussion

In view of increasing the usage of whole chickens, we explored consumers evaluations after cooking whole chicken dishes at their homes. Overall, the provided recipes/ dishes were predominantly rated positively in terms of taste and preparation, suggesting existing interest among participants. However, it should be noted that participants had been recruited based on their agreement to prepare whole chickens, and are therefore not representative of the general population. In the future a larger consumer survey is needed to estimate the proportion of “whole-chicken users”. Given this background, marketing strategies that target whole chickens directly to end customers, along with recipe suggestions or cooking bags/kits, are deemed particularly suitable. Households with medium to high net household income, families with children, and older individuals, in particular, appear to have a strong potential as consumer segments for whole chickens. These groups are more likely to engage in more complex cooking processes. Previous studies on the WTP for local and dual-purpose chickens also reported on the income relation, resulting that these groups should represent a significant portion of the targeted market section (Escobedo del Bosque et al., 2021; Gangnat et al., 2018; Makdisi and Marggraf, 2011). Moreover, participants who value regional origin in their food choices represent another relevant target group. Regionality was frequently mentioned as an important purchasing criterion and was found to correlate positively with household income. This is consistent with the German Nutrition Report 2024, in which 85 % of those over 60 years and 60 % of those aged 14-29 value regionality when shopping (BMEL, 2024).

In contrast, low-income households perceived the higher prices for a whole chicken as a huge barrier. The higher cost of local chicken breeds, due to extended rearing periods and elevated maintenance requirements, acts as a financial obstacle for these groups. These findings align with results from Depa et al. (2018), who reported that 49.4 % of low-income individuals in Germany relying on food banks were unable to access healthy, nutritious, and local food due to financial constraints. Additionally, younger participants without children in the household highlighted the greater preparation effort as deterrents. To address this barrier, marketing strategies could, for example, offer pre-seasoned whole chickens combined with ingredient boxes and easy-to-follow recipes, thereby reducing the expected preparation effort for especially younger consumers. At the same time, participants frequently expressed feelings of discomfort and even disgust, particularly when less processed whole chickens still retained visible animal features (e.g., blood, head, neck). The cognitive dissonance that arises when people enjoy eating meat while being reminded of its animal origin is described as the “meat paradox” and helps understanding these psychological barriers (Loughnan et al., 2014). Kunst and Hohle (2016) found empathy for animals greatly decreased when meat was highly processed. In our participant sample, feelings of disgust were reported more often by participants with an agricultural background, contrary to our expectation. Maybe this group tends to have a more realistic and emotionally complex relationship with animals and is more frequently confronted with slaughter for processing. Bock et al. (2007) observed that farmers often struggled when they described their emotional stance with regard to chickens in intensive poultry systems. It can be speculated that emotional detachment in intensive systems may serve as a coping mechanism to reduce guilt associated with animal processing and should be investigated in future research.

Another central aspect of the present study concerns the preparation process. Individuals with limited cooking skills particularly perceived the deboning of the chicken as challenging. In addition to the ingredients, further help with preparation could be provided (such as video tutorials). Further, the preparation time was also commonly reported as a hindrance herein. This corresponds with findings from the German Nutrition Report, where 56 % of respondents stated that speed and ease of preparation were within the top 3 most important criteria when choosing meals, indicating that complex and time-consuming preparation is a major barrier to consumption (BMEL, 2024).

These findings highlight the need for marketing strategies for whole chickens to be tailored not only by income levels but also by value orientations such as regional awareness and perceived effort. However, one structural barrier to market access lies not with the consumer but with the limited availability of local chicken breeds in conventional retail settings. The standardization of the poultry industry and its focus on a high-yielding hybrid lines restrict the accessibility of alternative breeds, even for motivated and willing consumers (Augère-Granier, 2019; DLG, 2024). Beyond domestic challenges, the findings also have implications in a broader, international context. The widespread preference for specific poultry cuts in the EU has serious consequences for global food markets. Thus, encouraging whole-animal consumption within Europe could not only reduce chicken dumping and promotes sustainable practices but also contributes to alleviating global inequalities and environmental burdens associated with the industrial meat trade (Pelikan et al., 2022).

Regarding sensory aspects, no significant differences were found between local chicken breeds and the commercial standard breed. This suggests that herein breed does not play a decisive role for palatability although the relatively low power regarding the breed effect needs to be acknowledged. Napolitano et al. (2013) found also that untrained consumers could not differentiate between different chicken breeds which is in line with a study on local chicken breeds and crossbreeds thereof in Germany (Escobedo del Bosque et al., 2022). Herein, subjective factors such as being familiar with the dish played a stronger role. Chicken soup, potentially due to simpler preparation and cultural familiarity, was rated more favorable than chicken fricassee in our study. Participants deviated from the recipe in about 40 % of cases by adding own spices. The so-called “IKEA effect” describes the tendency to value products more when one has actively contributed to their creation (Dohle et al., 2016; Radtke et al., 2019). Since more modifications occurred within the dish chicken soup than with the dish chicken fricassee, this may partly explain the higher ratings for the former. Notably, the method of removing meat from the bone had also a significant impact on participants' evaluations. Using own hands may lead to greater sensory engagement and control over texture and quality, which could enhance overall satisfaction (Font-I-Furnols and Guerrero, 2014). If the meat for the fricassee can be plucked unevenly by hand, frustration is reduced in case the removal of the meat by fork or knife should not work well.

The analysis of WTP showed that in addition to well-known factors such as income and price orientation, ethical considerations of regional origin play a role. Consumers’ buying decisions are strongly affected by the price of the item (BMEL, 2024; Escobedo del Bosque et al., 2021; Steenhuis et al., 2011). However, the study also revealed that interest in regional products does not automatically lead to purchasing behavior favoring local breeds. One core issue is a lack of consumer knowledge regarding dual-purpose breeds, agrobiodiversity, or the environmental impacts of industrial poultry production.

One limitation of this study is the convenience sampling of participants, who may already have a positive attitude towards using whole chickens. This bias is reflected in the sample segmentation, where the "positive attitude" group was dominant compared to segments marked by disgust or perceived effort. It remains to be shown through a larger consumer study how large this segment is in the general population. There, WTP may also be more heterogeneous than in our sample.

The German population was not represented by the sample since it leaned toward wealthier, educated people affecting generalizability. Social desirability bias along with a limited cultural diversity may also have influenced how the participants responded in the study.

Furthermore, the supplied chicken was frozen, and participants were not informed about this until pickup. Palatability of the chicken meat might have been affected since the defrosting process was not fully standardized. Furthermore, because the number of birds was limited (and bound to the overarching project), an incomplete-block design was used for the study. Hence, each participant evaluated only two chicken breeds thereby limiting sensory comparisons across all breeds.

## Conclusions

This study provided insights into opportunities and barriers for the use of whole chickens by private households in Germany. Overall, despite some openness for preparing whole chickens at home was observed, their wider adoption is limited by practical and psychological barriers, such as feelings of disgust or perceived effort. Hence, in order to successfully re-integrate whole chickens into the market, marketing strategies must extend beyond appeals to animal welfare or sustainability. It is vital that efforts are focused on reducing the workload for consumers when preparing meals. In order to achieve this, support must be provided for recipes and preparation behaviors. Familiar and culturally rooted recipes (e.g., chicken soup) are able to achieve a higher degree of acceptance. Furthermore, it is essential to ensure accessibility within conventional retail channels. Such comprehensive approaches are deemed necessary to realize the ecological and cultural potential of whole chickens from local breeds, thereby contributing to the preservation of agrobiodiversity.

## CRediT authorship contribution statement

**Claire Siebenmorgen:** Writing – original draft, Project administration, Methodology, Investigation, Conceptualization. **Annik Spreckelmeyer:** Writing – review & editing, Methodology, Investigation, Conceptualization. **Micha Strack:** Writing – review & editing, Visualization, Supervision, Formal analysis, Data curation. **Daniel Mörlein:** Writing – review & editing, Supervision, Resources, Project administration, Funding acquisition, Conceptualization.

## Disclosures

The authors declare no financial, professional, or personal relationships with people or organizations could influence the work reported within this paper.

## Figures and Tables

**Fig. A.2:**
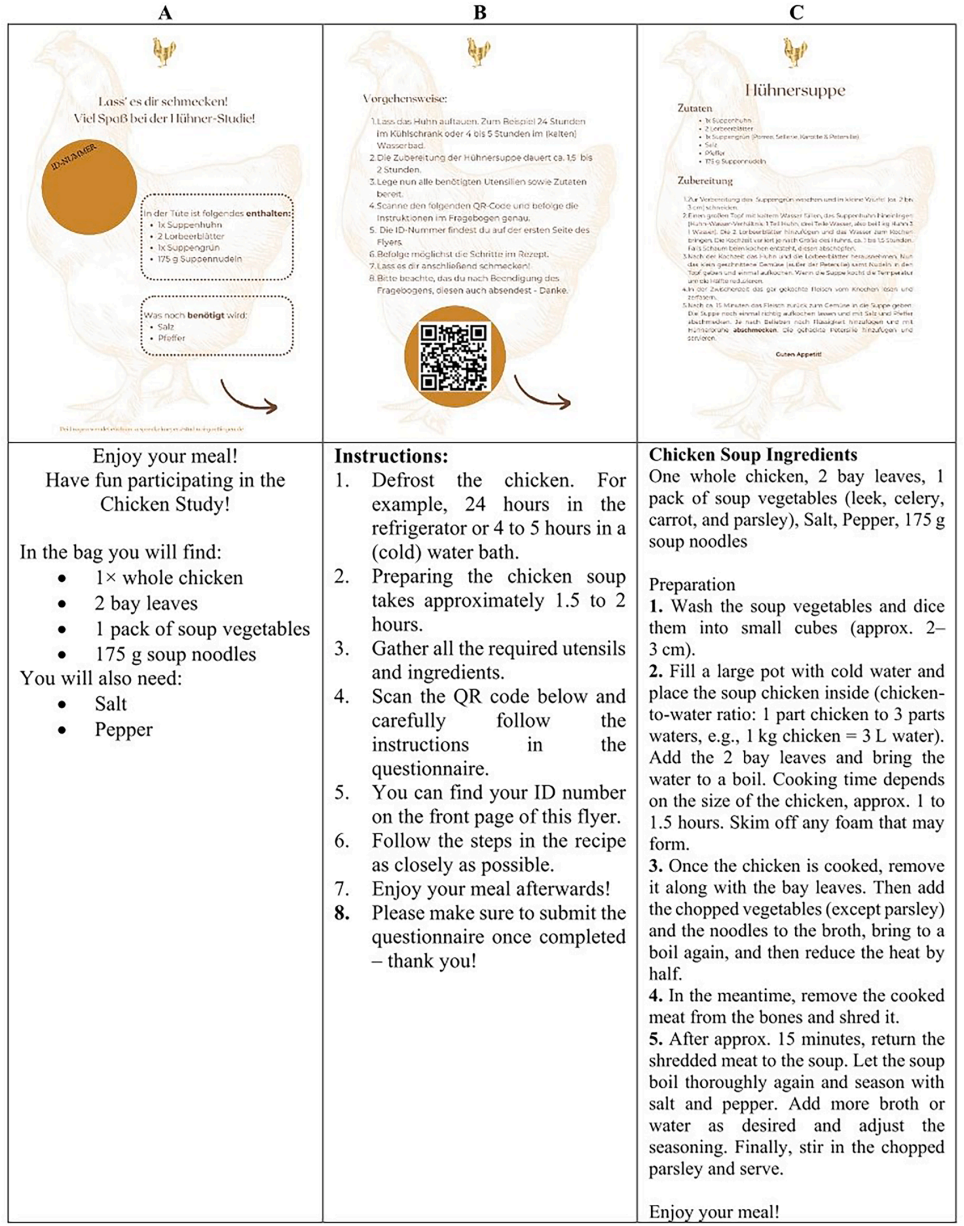
Recipe book for the chicken soup including (A) title page with information on the contents and ingredients that are still needed, (B) general procedure of the experiment, as well as QR code for the questionnaire, and (C) recipe with ingredients and step-by-step explanation.

**Table A.1:** Attitude questions.

Statement	Strongly agree	Agree	Neither agree nor disagree	Disagree	Strongly disagree
A whole chicken is too much meat for me/my household.	□	□	□	□	□
A whole chicken is too time-consuming to prepare.	□	□	□	□	□
A whole chicken allows for especially flavorful dishes.	□	□	□	□	□
A whole chicken is too fatty.	□	□	□	□	□
There are parts on a whole chicken that I/my household do not like.	□	□	□	□	□
A whole chicken is too expensive for me/my household.	□	□	□	□	□
A whole chicken reminds me too much of the living animal.	□	□	□	□	□
I find a whole chicken disgusting.	□	□	□	□	□
I find preparing a whole chicken too time-consuming for everyday cooking.	□	□	□	□	□
I consider dishes that use whole chickens easy to prepare.	□	□	□	□	□
The time required to prepare a whole chicken is too great for me.	□	□	□	□	□
I think too many utensils are needed to cook whole chickens.	□	□	□	□	□
Only a few ingredients are needed to prepare dishes with whole chicken.	□	□	□	□	□
